# *n*-type conversion of SnS by isovalent ion substitution: Geometrical doping as a new doping route

**DOI:** 10.1038/srep10428

**Published:** 2015-05-28

**Authors:** Fan-Yong Ran, Zewen Xiao, Yoshitake Toda, Hidenori Hiramatsu, Hideo Hosono, Toshio Kamiya

**Affiliations:** 1 Materials and Structures Laboratory, Tokyo Institute of Technology, 4259 Nagatsuta, Midori-ku, Yokohama 226-8503, Japan; 2 Materials Research Center for Element Strategy, Tokyo Institute of Technology, 4259 Nagatsuta, Midori-ku, Yokohama 226-8503, Japan; 3 Frontier Research Center, Tokyo Institute of Technology, 4259 Nagatsuta, Midori-ku, Yokohama 226-8503, Japan

## Abstract

Tin monosulfide (SnS) is a naturally *p*-type semiconductor with a layered crystal structure, but no reliable *n*-type SnS has been obtained by conventional aliovalent ion substitution. In this work, carrier polarity conversion to *n*-type was achieved by isovalent ion substitution for polycrystalline SnS thin films on glass substrates. Substituting Pb^2+^ for Sn^2+^ converted the majority carrier from hole to electron, and the free electron density ranged from 10^12^ to 10^15^ cm^−3^ with the largest electron mobility of 7.0 cm^2^/(Vs). The *n*-type conduction was confirmed further by the position of the Fermi level (*E*_F_) based on photoemission spectroscopy and electrical characteristics of pn heterojunctions. Density functional theory calculations reveal that the Pb substitution invokes a geometrical size effect that enlarges the interlayer distance and subsequently reduces the formation energies of Sn and Pb interstitials, which results in the electron doping.

Control of carrier polarity conversion in semiconductor is important to produce high-performance semiconductor devices such as solar cells and light emitters, and is actually utilized in conventional semiconductors such as Si and compound semiconductors. On the other hand, it is known that such bipolar doping is not attained easily in other semiconductors; e.g., most of oxide semiconductors are of naturally *n*-type, and it is difficult to obtain *p*-type conduction in the same materials as known e.g. for SnO_2_, and vice versa e.g. for Cu_2_O. To date, several, but a limited number of doping routes have been recognized and employed. For ionic semiconductors, aliovalent ion substitution and off-chemical stoichiometry are known well; e.g., substitution of Zn^2+^ with Ga^3+^ increased the electron density in ZnO^1^, and Cu vacancy increased the hole density in Cu_2_O^2^. Further, H doping is now recognized as an important and effective route for *n*-type doping in oxide semiconductors^3^. For organic semiconductors and devices, chemical doping, which is caused by partial charge transfer originating from different electron affinities of constituent atoms/functional groups, is important^4^. The most popular route for carrier polarity conversion is aliovalent ion substitution; actually, *n*-type conversion of SnO was realized by substituting Sb^3+^ ions for the Sn^2+^ ions^5^. However, up to now, this route has not been succeeded in many semiconductors, such as SnS.

SnS is a naturally *p*-type semiconductor with hole densities of 10^15^–10^18^ cm^−3^ and the high mobilities ~90 cm^2^/(Vs)^6^,^7^. It has a layered crystal structure along the *a*-axis direction as shown in Fig. 1a, which belongs to the orthorhombic lattice (the space group *Pnma*, No. 62). Due to its reasonable small bandgap of ~1.07 eV^8^ and strong optical absorption coefficients above the bandgap (>10^5^ cm^−1^), SnS is expected to be a promising absorber material for low-cost thin-film solar cells. Thus, numerous *n*-type materials, including CdS^9^,^10^, SnS_2_^11^, FeS_2_^12^, TiO_2_^13^, ZnO^14^, and amorphous- Si^15^, have been employed for fabricating heterojunction SnS-based solar cells. However, the highest energy conversion efficiency reported up to now is limited to ~4%^16^,^17^, which is much lower than the theoretically-predicted value of 24%^18^. The low efficiency might suffer from the unfavorable band alignments and the large lattice mismatches in the heterojunction structures^19^,^20^. Fabricating a homojunction solar cell with *p*-SnS/*n*-SnS structure would solve this problem.

With this line, much effort has been devoted to obtaining *n*-type SnS materials by substituting the Sn^2+^ ions with aliovalent ions with the charge state of 3+. Dussan *et al.* report that Bi^3+^-doped SnS exhibits *n*-type conduction when the Bi concentration is larger than 50%^21^. Whereas, a Bi_2_S_3_ impurity phase, which is also *n*-type, was observed in their heavily Bi-doped SnS films^22^. Sajeesheesh *et al.* claim that *n*-type SnS thin films are obtained by chemical spray pyrolysis, but their result might be due to a significant *n*-type Sn_2_S_3_ impurity phase in the films^23^. Very recently, Sinsermsuksakul *et al.* tried to obtain *n*-type SnS by Sb^3+^ doping; however, except for great increase in the electrical resistance of the SnS film, no *n*-type conduction was observed^24^. That is, no reliable *n*-type SnS material has yet been reported.

In this work, we succeeded in fabricating reliable *n*-type SnS films by isovalent Pb^2+^ doping. We found that the doping mechanism is strikingly different from the conventional doping routes such as ion substitution, off-stoichiometry, and chemical doping. Substitution for the Sn^2+^ ion with a larger Pb^2+^ ion increases the interlayer distance in SnS, and this geometrical effect induces the formation of Sn/Pb interstitials easier, and the interstitial ions work as donors.

We deposited 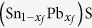 films (*x*_*f*_: film chemical composition) by pulsed laser deposition (PLD) on SiO_2_ glass substrates in a H_2_S gas flow to control the chemical stoichiometry ([Sn] + [Pb]): [S] (the parentheses denote the concentrations), where the H_2_S pressure (*P*) was a variable parameter. 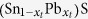 polycrystalline disks with the target chemical composition *x*_*t*_ = 0.18, 0.37, and 0.66 were used as ablation targets. X-ray fluorescence (XRF) spectroscopy confirmed that these *x*_*t*_ produced thin films with *x*_*f*_ = 0.08–0.5. The details of experimental and calculation are found in method part of this paper.

## Results and discussion

**Figure 1:**
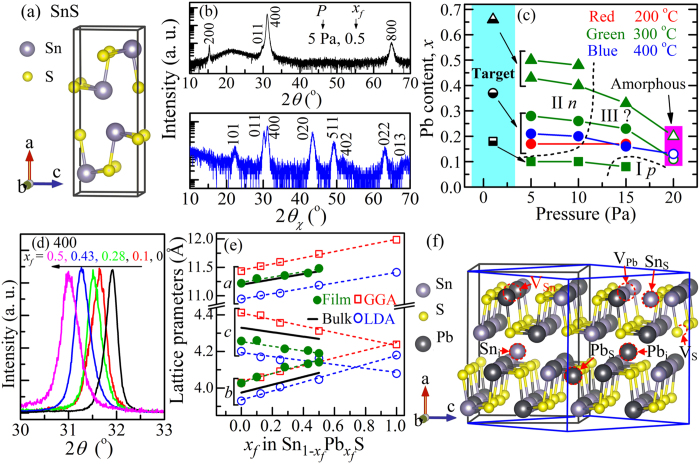
Structure of 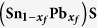 films. (**a**) Crystal structure of pure SnS. (**b**) Out-of-plane (top panel) and in-plane (bottom panel) synchronous scan XRD patterns of 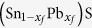 film with *x*_*f*_* = *0.5 grown at 300 °C and 5 Pa. (**c**) Pb content (*x*_*t*_ for target, *x*_*f*_ for thin films) as a function of pressure, substrate temperature, and *x*_*t*_. The half-filled symbols indicate the targets, the closed circles the orthorhombic phase crystalline films, and the open symbols amorphous films. (**d**) 400 out-of-plane XRD diffraction peaks of the 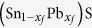 films grown at 300 °C with various *x*_*f*_ values. (**e**) Lattice parameters (*a*, *b*, *c*) of 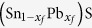 films as a function of *x*_*f*_. The closed circles indicate those obtained with the thin films, the solid lines are those of bulk (Sn_1–*x*_Pb_*x*_)S taken from ref. 25, and the open symbols are the calculation results obtained by DFT in this work. The dashed straight lines are guides for eyes. (**f**) (Sn_1-*x*_Pb_*x*_)S supercell model used for DFT calculations. The black line box draws the (Sn_16–*n*_Pb_*n*_)S_16_ supercell model used for calculating the lattice parameters in Fig. 1e. The blue line box draws the (Sn_16_Pb_16_)S_32_ supercell model used for defect calculations in Fig. 4, where the intrinsic defect models examined in this study are indicated also in the figure.

First, we confirmed the film structures by X-ray diffraction (XRD). Figure 1b shows a typical out-of-plane 2*θ*/*ω* synchronous scan (top panel) and an in-plane synchronous 2*θ*_χ_/*φ* scan (bottom panel) XRD patterns of the 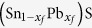 film with *x*_*f*_* = *0.5 grown at substrate temperature (*T*_s_) = 300 °C and *P* = 5 Pa. The out-of-plane XRD pattern exhibited strong 200, 400 and 800 diffractions of the orthorhombic structure, which is the same as that of pure SnS in Fig. 1a, along with a weak 011 diffraction. As seen in Figure S1 (supplementary information), orthorhombic 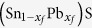 films were obtained at *P* ≤ 15 Pa (corresponding to the closed symbols in Fig. 1c); while, amorphous films were obtained when *P* was increased to 20 Pa (the open symbols in Fig. 1c). The in-plane synchronous 2*θ*_χ_/*φ* scan (bottom panel) shows powder-like patterns with all possible *hkl* diffractions, suggesting that the film did not have in-plane orientation. It was further confirmed by in-plane rocking patterns (*φ* scan at fixed 2*θ*_χ_, data not shown); all the data showed that the crystallized films did not have a preferential orientation in plane. These results indicate that the 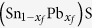 films were polycrystalline films with a strong 100 preferential orientation normal to the substrate. No impurity phase was detected both in the out-of-plane and the in-plane XRD patterns.

Figure 1c shows the variation of *x*_*f*_ as functions of *P*, *T*_s_ and *x*_*t*_. It is seen that all the *x*_*f*_ values were smaller than the *x*_*t*_ values of the corresponding targets, and the *x*_*f*_ values decreased with increasing *P*. As seen for the target with *x*_*t*_ = 0.37, the maximum amount of Pb was incorporated when the films were grown at *T*_*s*_ = 300 °C. We, therefore, employed *T*_s_ = 300 °C hereafter.

**Figure 2:**
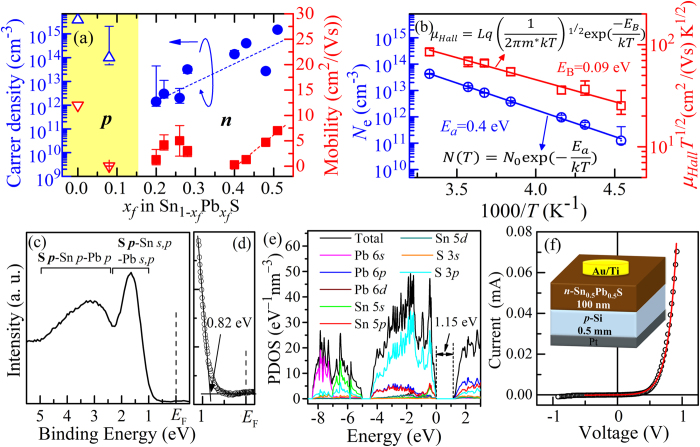
Electrical and electronic properties. (**a**) Carrier density and mobility of 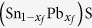 films as a function of *x*_*f*_ measured by Hall effect. (**b**) Temperature dependences of electron density (*N*_e_, blue line) and mobility (*μ*_e_, red line) of *n*-type 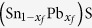 film with *x*_*f*_* = *0.48. (**c**,**d**) UPS spectrum of *n*-type (Sn_0.5_Pb_0.5_)S film. (**d**) shows a magnified view near *E*_F_. (**e**) Projected DOS of (Sn_0.5_Pb_0.5_)S calculated by DFT with GGA functionals. (**f**) I-V characteristics of *n*-(Sn_0.5_Pb_0.5_)S/*p*-Si pn heterojunction. Inset shows the device structure.

The crystallized region in Fig. 1c is classified further to three regions as indicated by the dashed lines. Region I is “*p*-type region” (high *P* ≥ 15 Pa at low *x*_*f*_ < 0.1), where the films still exhibited *p*-type conduction with low hole densities (*N*_h_) and low hole mobilities (*μ*_h_) (measured by Hall effect, details will be discussed for Fig. 2). *n*-type 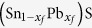 films were obtained in Region II (“*n*-type region”, *x*_*f*_ ≥ 0.15 at low *P* ≤ 10 Pa). The electron density (*N*_e_) and mobility (*μ*_e_) changed largely with *x*_*f*_ and *P*, which will be discussed later on. Region III is the intermediate region (“highly-resistive region”, low *x*_*f*_ & low *P*, and high *x*_*f*_ & high *P*), where the films exhibited very high resistivity >10^5^ Ω·cm, and the Hall effect measurements did not give definite Hall voltage signs.

Here, we discuss the doping structure of Pb. Figure 1d shows the variation of the out-of-plane 400 diffraction angles 2*θ*_400_ obtained by 2*θ*/*ω* synchronous scan as a function of *x*_*f*_. The 2*θ*_400_ value shifted to lower angles as *x*_*f*_ increased, indicating that the *a*-axis expanded with increasing *x*_*f*_. The lattice parameters obtained from the out-of-plane 400 and the in-plane 020 and 011 diffraction angles are summarized as a function of *x*_*f*_ in Fig. 1e. As *x*_*f*_ increased from 0 to 0.5, the *a* and *b* values increased linearly from 1.12 to 1.14 nm and from 0.403 to 0.414 nm, respectively, whereas the *c* value decreased from 0.426 to 0.419 nm; i.e., the interlayer distance (corresponding to the *a* value) increased. The solid lines in Fig. 1e represent the lattice parameters of the(Sn_1–*x*_Pb_*x*_)S bulk sample reported by Leute *et al.*^25^. The *a* values of our films are almost the same as those of the bulk samples. However, the *b* and *c* values exhibited non-negligible deviations from the bulk values; *i.e.*, the *b*-axis was expanded while the *c*-axis shrunken compared from the bulk values. The reason is not clear, but defects in the polycrystalline films would cause the structural difference. Figure 1e also compares the variation of the lattice parameters with those obtained by density functional theory (DFT) calculations (the open symbols) performed with the (Sn_16–*n*_Pb_*n*_)S_16_ supercell model (Pb substitution model) indicated by the black line box in Fig. 1f. Here, local density approximation (LDA) and generalized gradient approximation (GGA) functionals are compared. As will be seen later, GGA provides better description about the electronic structure; however, here we can see that the experimental results for the Pb substitution model were within the variation of the functionals (typically, the ground-state lattice parameters by DFT include errors within 2–3%). That is, this model, where the Sn sites are substituted by Pb, explains the experimental structure well, and strongly supports that the Pb dopants are successfully incorporated to the Sn sites in the SnS lattice. We also confirmed that the films are uniform in microstructures and chemical compositions, and no segregation (e.g., a Pb-rich impurity phase) was detected by atomic force microscopy (AFM), field-emission scanning electron microscopy (FE-SEM), and electron-probe microanalysis (EPMA) (supplementary information Figure S2 and Table S1).

Figure 2a shows Hall effect measurement results as a function of *x*_*f*_. The pure SnS film showed *p*-type conduction with *N*_h_ ~ 4.1 × 10^15^ cm^−3^ and *μ*_h_ ~ 12 cm^2^/(Vs). The 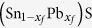 film with *x*_*f*_  = 0.08 fabricated at *P = *15 Pa still showed *p*-type conduction but with the low *N*_h_ ~ 1.0 × 10^14^ cm^−3^ and the very small *μ*_Hall_ in the order of 10^−2^ cm^2^/(Vs). When *x*_*f*_ ≥ 0.2, *n*-type conductions were observed for the films fabricated at *P = *5 and 10 Pa. For the *n*-type 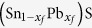 film with *x*_*f*_ = 0.2, *N*_e_ and *μ*_Hall_ were 1.4 × 10^12^ cm^−3^ and 1.3 cm^2^/(Vs), respectively. *N*_e_ increased with increasing *x*_*f*_ and reached 2.0 × 10^15^ cm^−3^ for *x*_*f*_* = *0.5. *μ*_e_ was not changed largely when *x*_*f*_ < 0.3 (*N*_e_ < 3.2 × 10^13^ cm^−3^). At *x*_*f*_ values >0.4, *μ*_e_ increased almost linearly, and the maximum value of 7.0 cm^2^/(Vs) was obtained for *x*_*f*_  = 0.5.

Figure 2b shows temperature dependences of *N*_e_ and *μ*_e_ for the 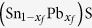 film with the room-temperature *N*_e_ of 4.3 × 10^13^ cm^−3^ (*x*_*f*_* = *0.48 grown at 10 Pa). The *N*_e_ shows a thermally-activated behavior with an active energy of *E*_a_ ~ 0.4 eV. From a simple approximation in the impurity region *N*_e_ = (*N*_D_*N*_C_)^1/2^exp[–(*E*_C_ – *E*_D_)/(2*k*_*B*_T)] (*N*_D_ is the donor density, *N*_C_ the conduction band effective density of states (DOS), *E*_C_ – *E*_D_ the donor level measured from the conduction band minimum *E*_C_, *k*_*B*_ the Boltzmann constant), *E*_C_ – *E*_D_ and *N*_D_ are estimated to be ~0.8 eV and 2.5 x 10^21^ cm^−3^ (*N*_C_ = 2.8 x 10^19^ cm^−3^ is taken from Si), respectively. More accurate estimation was performed based on the total DOS obtained by the DFT calculation and the semiconductor statistics^26^, which provided *E*_C_ – *E*_D_ = 0.75 eV, *E*_C_ – *E*_F_ = 0.30 eV, and *N*_D_ = 1.0 × 10^21^ cm^−3^, agreeing well with the above simple estimation and guaranteeing that the film is in the impurity region in this measurement temperature range. On the other hand, although *E*_F_ was closer to *E*_C_ as in usual *n*-type semiconductors, the donor level *E*_D_ was closer to the valence band maximum energy (*E*_V_) rather than *E*_C_, showing that the *n*-type doping in the 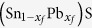 films is a bit different from the usual *n*-type semiconductors.

As shown by the red line in Fig. 2b, *μ*_e_ decreased with decreasing the temperature, and the ln(*μ*_Hal_*T*^1/2^)–*T*^*−*1^ plot exhibited a good straight line in the whole *T* range, suggesting that the electron transport in the film was dominated by grain boundary (GB) potential barriers as proposed by Seto *et al.*^27^, where electron transport is disturbed by potential barriers formed due to the electrons trapped at acceptor-type defects at the GBs. The GB potential barrier height *E*_B_ is estimated to be approximately 0.09 eV (the equation is given in Fig. 2b^27^). From this result, we can estimate the potential electron mobility *μ*_0_ (i.e., the ideal value when no GB affects the carrier transport) by extrapolating *E*_B_ to zero (*i.e.*, *μ*_0 = _*μ*_Hall_ exp(*E*_B_/*kT*)), which gives *μ*_0_ ~ 1.6 × 10^2^ cm^2^/(Vs).

**Figure 3:**
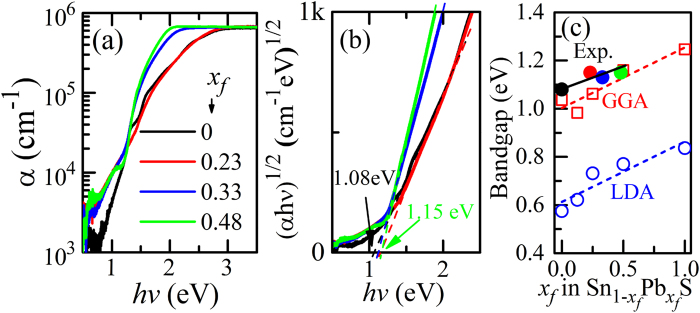
Optical properties. (**a**) Typical optical absorption spectra and (**b**) (αhν)^1/2^ – hν plots (indirect-transition model) of 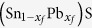 films with various *x*_f_ (the *x*_*f*_ values are indicated in the figure (**a**)). The values in (**b**) indicate the optical bandgaps obtained from the straight regions in the (αhν)^1/2^ – hν plots. (**c**) Variation of optical bandgaps with *x*_*f*_. Those calculated by DFT with LDA and GGA functionals are also shown.

Figure 2c shows a valence band structure of a (Sn_0.5_Pb_0.5_)S film measured by ultraviolet photoemission spectroscopy (UPS). A sharp peak at 1–2 eV and a broad peak at 2.5–4.5 eV can be observed, agreeing with the projected DOS (PDOS) calculated by DFT in Fig. 2e. The valence band consists mainly of S 3*p* orbitals, which slightly hybridized with Sn 5*s*, Sn 5*p*, Sn 5*d*, Pb 6*s*, Pb 6*p*, and Pb 6*d* orbitals. As seen in Fig. 2d, the observed *E*_F_ of the (Sn_0.5_Pb_0.5_)S film is located at 0.82 eV above VBM. From the bandgap value of 1.15 eV (will be discussed for Fig. 3), the *E*_C_ – *E*_F_ value is estimated to be 0.33 eV, closer to conduction band minimum (CBM).

To further confirm the *n*-type conduction of these films, *n*-type (Sn_0.5_Pb_0.5_)S/*p*-type Si pn heterojunction was prepared (the device structure is shown in the inset to Fig. 2f). The *n*-(Sn_0.5_Pb_0.5_)S film and the *p*-Si wafer used had *N*_e_ = 2 × 10^15^ and *N*_h_ = 5 × 10^15^ cm^−3^, respectively. The current–voltage (*I*–*V*) characteristic of the pn junction (Fig. 2f) showed a clear rectifying characteristic, further supporting the *n*-type conduction of the 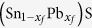 film. The band alignment of this pn heterojunction (supplementary information Figure S3) gives the built-in potential (*V*_bi_) of 0.76 eV. This *V*_bi_ roughly explains the experimental threshold voltage of the pn heterojunction ~0.67 V obtained by extrapolating the straight line region in Fig. 2f.

Figures 3a and b show typical optical absorption spectra and (α*h*ν)^1/2^ – *h*ν plots (the indirect-transition model) of the 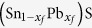 films fabricated at the various conditions, respectively. The pure SnS film (the black line in (a)) exhibited very weak subgap absorption, and the bandgap estimated from the (α*h*ν)^1/2^–*h*ν plot is 1.08 eV, agreeing well with the literature theoretical value of ~1.07 eV^8^. The bandgaps estimated from (b) are shown in Fig. 3c as a function of *x*_*f*_, showing that the bandgap value increased with *x*_*f*_. Comparing with the calculated bandgap values, it is concluded that the GGA functional reproduces the experimental values better than LDA.

Here, we like to discuss the origin of the *n*-type doping in the 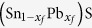 films. It is known that Pb ions favor to take +2 and +4 oxidation states, and the latter would explain the *n*-type doping if Pb^4+^ substitutes the Sn^2+^ site. However, the above DFT calculations for the (Sn_32–*n*_Pb_*n*_)S_32_ supercell models indicated that the Pb substitutions at the Sn site (denoted Pb_Sn_) generate no free charges because the Pb is ionized to Pb^2+^. We also confirmed by X-ray photoemission spectroscopy (XPS) that the calibrated energy level of Pb 4f_7/2_ in the 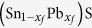 with *x*_*f*_  = 0.5 was 137.55 eV and close to that in a reference PbS (137.15 eV), which supports that the oxidation state of the Pb incorporated in the 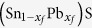 films is +2 (supplementary information Figure S4). This result in turn indicates that the doping mechanism by this Pb substitution is not an aliovalent ion substitution, and the conventional substitution models do not explain the *n-*type doping in the 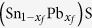 films.

**Figure 4:**
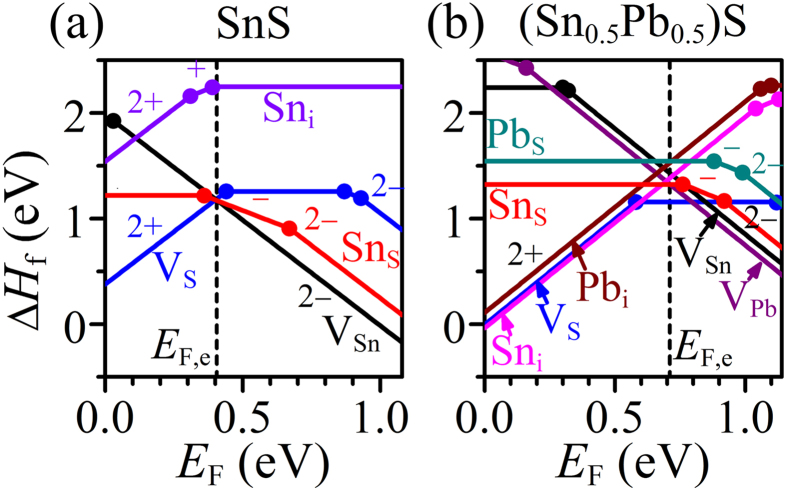
Formation enthalpies *ΔH*_f_ of intrinsic defects calculated at S-poor limit. Calculated *ΔH*_f_ for (**a**) pure SnS and (**b**) (Sn_16_Pb_16_)S_32_ models as a function of *E*_F_ at S-poor limit. The values in the figures represent the charge states of the defects in the DFT calculations. The black dashed lines represent the equilibrium *E*_F_ (*E*_F,e_) calculated self-consistently.

Here, we discuss the microscopic mechanism of the *n*-type doping by the Pb substitution. Firstly, we should remind that the *n*-type conduction was obtained only when a film was grown under the S-poor condition (*i.e.*, at low *P*). We calculated the formation enthalpies (Δ*H*_f_) of intrinsic defects in the pure SnS and the (Sn_0.5_Pb_0.5_)S models (the blue line box in Fig. 1f) under the S-poor limit condition as a function of *E*_F_ by DFT calculations as shown in Fig. 4a and b, respectively. Vacancies (V_S_, V_Sn_, V_Pb_), anti-site defects (Sn_S_, Pb_S_), interstitials (Sn_i_, Pb_i_) were examined (see Fig. 1f for the models) with the defect charge states from 2+ to 2−. These calculations employed LDA functionals not GGA in order to compare with the previously-reported results for pure SnS by Vidal *et al.*.^28^ The present result of the pure SnS model (Fig. 4a) is almost the same as their results; *i.e.*, the most stable charge state of V_S_ transits from 2+ to 0 at *E*_F_ ~ 0.4 eV, corresponding to the charge transfer energy level of *ε*_2+/0_. The most stable defect changed from V_S_^2+^ to Sn_S_^−^ & V_Sn_^2−^ at *E*_F_ ~ 0.4 eV. As V_S_^2+^ acts as a doubly-ionized donor while Sn_S_^−^ and V_Sn_^2−^ are ionized acceptors, suggesting that SnS is intrinsically a compensated *p*-type semiconductor. For quantitative analysis, the equilibrium *E*_F_ (*E*_F,e_) at 400 °C (i.e., we assume the defect structures at the growth temperature were frozen to room temperature) was calculated by considering all the *ΔH*_*f*_ values and the semiconductor statistics, giving *E*_F,e_ – *E*_V_ = 0.41 eV with [V_S_^2+^] = 5.0 × 10^15^, [V_Sn_^2−^] = 3.9 × 10^15^, and [Sn_S_^−^] = 5.2 × 10^15^ cm^−3^ for the SnS model. This means that the free electrons were generated from V_S_^2+^ at 1.0 × 10^16^ cm^−3^ but compensated by larger amounts of holes generated from V_Sn_^2−^ and Sn_S_^−^ at 1.3 × 10^16^ cm^−3^, resulting in the *p*-type conduction. It should be noted that the Sn_i_ has a very large *ΔH*_f_ and is not likely formed in pure SnS.

For the (Sn_0.5_Pb_0.5_)S model in Fig. 4b, although the Δ*H*_f_ values of V_S_^2+,0^ remained unchanged, that of Sn_i_^2+^ was reduced and that of V_Sn_^2−^ increased significantly compared to those in the pure SnS, which is because the interlayer distance (corresponding to the *a*-axis length) and the *b*-axis lattice parameters were increased by the Pb substitution (as also observed experimentally in Fig. 1e). Similar *ΔH*_f_ behaviors were found also for Pb_i_^2+^ and V_Pb_^2−^, respectively. That means, this geometrical alternation makes the generation of the donor Sn_i_^2+^ and Pb_i_^2+^ easier whereas suppresses the generation of the acceptor V_Sn_^2−^ and V_Pb_^2−^, suggesting *n*-type doping. The *E*_F,e_ calculation at 300 °C gave *E*_F,e_ – *E*_V_ = 0.71 eV with [V_Sn_^2−^] = 5.5 × 10^13^, [V_Pb_^2−^] = 4.6 × 10^14^, [Sn_i_^2+^] = 6.1 × 10^14^, and [Pb_i_^2+^] = 3.2 × 10^13^ cm^−3^. Note that the V_S_ has the charge neutral state at this *E*_F,e_, and does not contribute to carrier doping. Consequently, the free holes were generated at 1.0 × 10^15^ cm^−3^, while the larger amounts of free electrons were generated at 1.3 × 10^15^ cm^−3^, resulting in *n*-type doping. Finally, we conclude that Sn_i_ and Pb_i_ are the most plausible origin of the *n*-type conduction in the 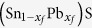 films.

## Summary

In summary, *n*-type conduction in SnS was achieved by isovalent Pb substitution with the maximum electron mobility of 7 cm^2^/(Vs). DFT calculations proposed a new doping model where the Pb substitution at the Sn sites induces the formation of Sn_i_ and/or Pb_i_ and produces donors. To date, carrier polarity control in semiconductor is achieved mainly by aliovalent ion substitution, off chemical stoichiometry, chemical doping and so on. This work revealed that substitution by an isovalent ion can also induce carrier doping by a two-step indirect mechanism through a geometrical effect and subsequent formation of charged defects.

The present finding provides a novel idea for carrier doping. Even keeping the same crystal structure and the ion charges, easiness of impurity doping, in particular for atoms/ions with largely-different sizes, depends significantly on the lattice parameters and the internal atomic coordinates, which can be altered also by impurity doping. Further, although substitution doping usually requires aliovalent ion doping to alter the carrier polarity or concentration, geometrical doping has more flexibility because isovalent ion doping would also work for carrier doping.

This way of thinking would provide more flexibility to explore new doping routes, open a new way for controlling carrier polarity and density in novel semiconductors in which conventional aliovalent ion substitution is difficult.

## Methods

### Film fabrication


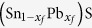
 films of 100–200 nm in thickness were grown on SiO_2_ glass substrates by pulsed laser deposition (PLD) using a KrF excimer laser (248 nm in wavelength, 3–6 J/cm^2^ of laser energy density, and 10 Hz of repetition rate) with 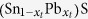 polycrystalline targets in a H_2_S gas flow to control the S stoichiometry. The base pressure of the growth chamber was 1 × 10^−5^ Pa. *T*_s_ was varied from 200 to 400 °C, and *P* of an Ar/H_2_S mixing gas (80/20%) from 5 to 20 Pa.

### Characterization

The crystalline phase and crystal structure of the obtained films were characterized by X-ray diffraction (XRD, radiation source = Cu Kα). Optical properties were obtained by measuring transmittance (*T*_r_) and reflectance (*R*) spectra. The absorption coefficient (*α*) was estimated by *α* = ln[(1−*R*)/*T*_r_]/*d*, where *d* is the film thickness. Electrical properties of the SnS films were analyzed by Hall effect measurements using the van der Pauw configuration with an AC modulation of magnetic field. The Pb content in the films (*x*_*f*_) were determined by X-ray fluorescence (XRF) spectroscopy calibrated by the chemical compositions obtained by inductively-coupled plasma-atomic emission spectroscopy (ICP-AES). The valence band structures were observed by UPS (excitation source = He I, 21.2 eV), where the films were protected in an Ar atmosphere during the transfer from the PLD chamber to the UPS chamber. The oxidation state of Pb was examined by x-ray photoemission spectroscopy (XPS, Mg Kα).

### Calculation

Stable crystal/defect structures, their electronic structures, and formation energies of intrinsic defects were calculated by density functional theory (DFT) calculations with local density approximation (LDA) and generalized gradient approximation (GGA) PBE96 functionals using the Vienna Ab initio Simulation Package (VASP 5.3.3)^29^. The plane wave cutoff energy was set to 323.3 eV. A 32-atoms supercell model ((Sn_16–*n*_Pb_*n*_)S_16_, black line in Fig. 1f) and a 4 × 6 × 5 *k*-mesh were used for the calculations of structural properties and electronic structures. The defect calculations were performed using a 64-atoms model ((Sn_16_Pb_16_)S_32_, blue line in Fig. 1f) and a 3 × 3 × 3 *k*-mesh. The procedure for calculating the defect Δ*H*_f_ along with the general corrections followed the methodology reviewed by Zunger *et al.*^30^,^31^. The equilibrium Fermi levels (*E*_F,e_) were determined using the calculated density of states (DOS) by solving semiconductor statistic equations self-consistency so as to satisfy the charge neutrality condition^32^.

## Additional Information

**How to cite this article**: Ran, F.-Y. *et al.* *n*-type conversion of SnS by isovalent ion substitution: Geometrical doping as a new doping route. *Sci. Rep.* **5**, 10428; doi: 10.1038/srep10428 (2015).

## Supplementary Material

Suplementary Information
