# Supplementary material for: n-type conversion of SnS by isovalent ion substitution: Geometrical doping as a new doping route
Source: Sci Rep. 2015 May 28;5:10428. doi: 10.1038/srep10428 (PMC4446993; doi:10.1038/srep10428)
Supplement: Suplementary Information [file srep10428-s1.pdf]

n-type conversion of SnS by isovalent ion substitution: Geometrical doping as a new doping route

Fan-Yong Ran[1], Zewen Xiao[1], Yoshitake Toda[3], Hidenori Hiramatsu[1,3], Hideo Hosono[1,2,3], and Toshio Kamiya[1,3,*]

1. Materials and Structures Laboratory, Tokyo Institute of Technology, 4259 Nagatsuta, Midori-ku, Yokohama 226-8503, Japan.
2. Frontier Research Center, Tokyo Institute of Technology, 4259 Nagatsuta, Midori-ku, Yokohama 226-8503, Japan.
3. Materials Research Center for Element Strategy, Tokyo Institute of Technology, 4259 Nagatsuta, Midori-ku, Yokohama 226-8503, Japan.

*Corresponding Author. Electronic mail: kamiya.t.aa@m.titech.ac.jp.

Supplementary Information

1. XRD patterns of $(Sn_{1-x_f}Pb_{x_f})S$ films.

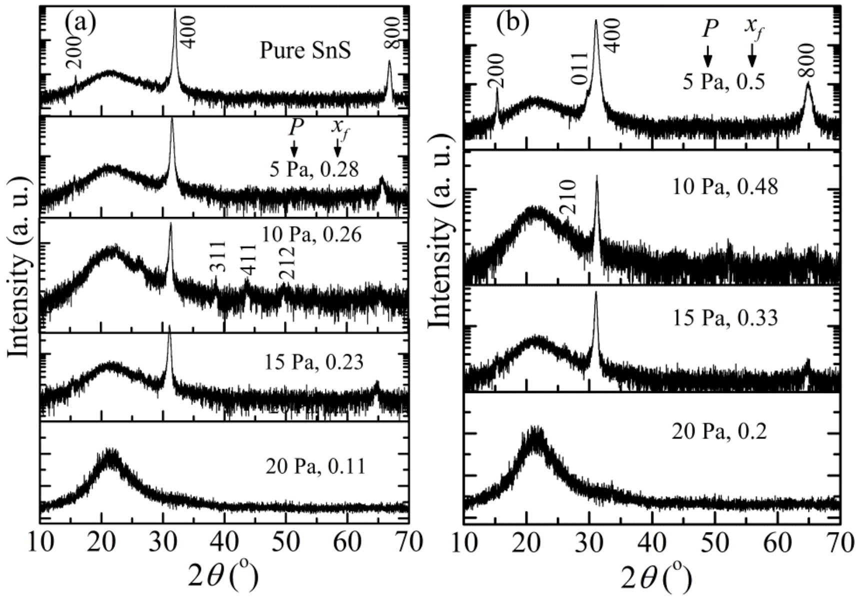

Figure S1 | XRD patterns. (a) and (b) XRD patterns of pure and $(Sn_{1-x_f}Pb_{x_f})S$ films fabricated at 300 ^{o}C at various conditions.

Typical out-of-plane XRD patterns of $(Sn_{1-x_f}Pb_{x_f})S$ films fabricated at various deposition conditions are shown in **Figure S1**. The XRD pattern of a pure SnS film is also shown at the top panel of **Figure S1**a for comparison. The XRD patterns of the films grown at $P \leq 15$ Pa exhibited strong 200, 400 and 800 diffractions from the orthorhombic structure of SnS. Whereas, no obvious diffraction was observed for the films grown at 20 Pa, indicating the films were amorphous.

2. Atomic force microscopy (AFM) and field-emission scanning electron microscopy (FE-SEM) images of ($Sn_{1-x_f}Pb_{x_f}$)S films.

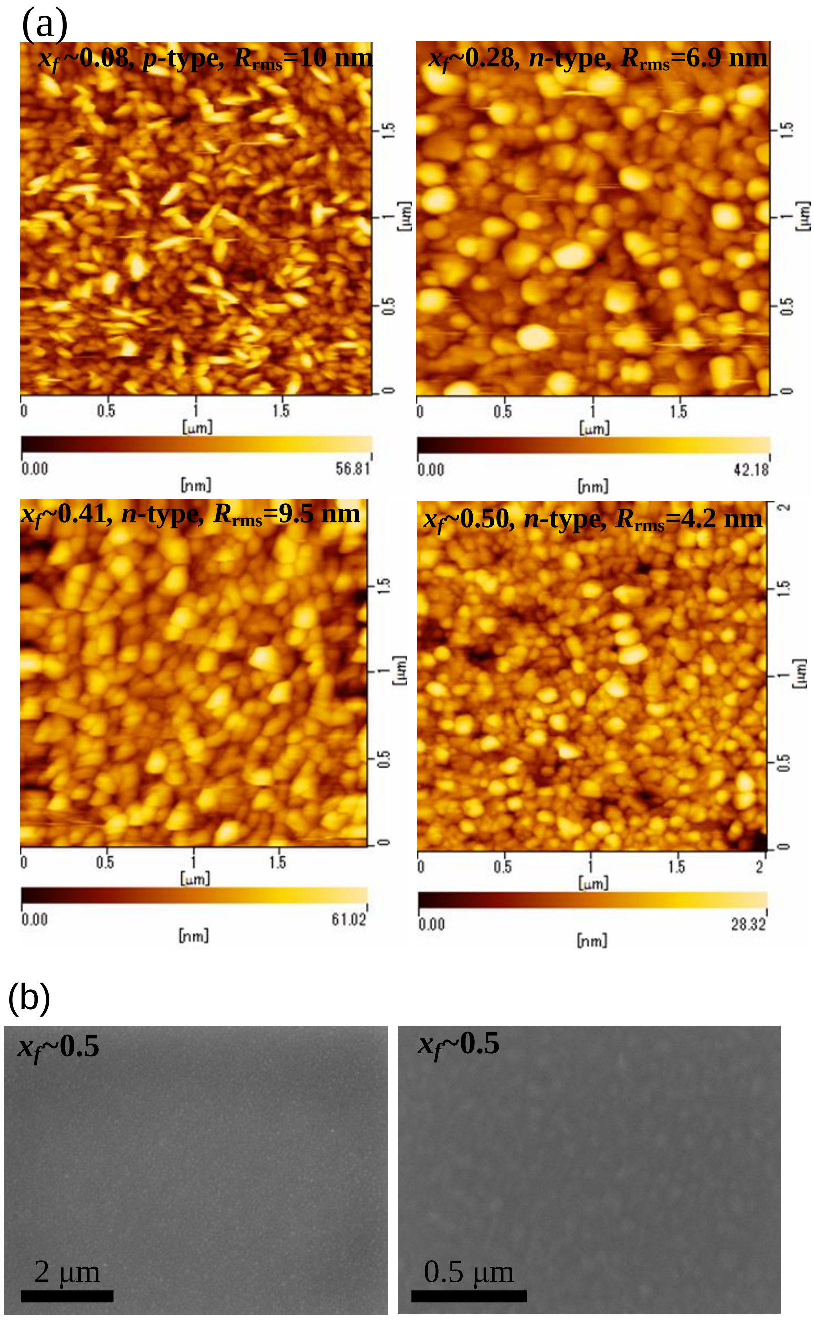

Figure S2 | Surface morphology. (a) AFM images of ($Sn_{1-x_f}Pb_{x_f}$)S films with different x_f values. (left-top) $x_f \sim 0.08$, p-type, R_{rms} (root mean square surface roughness) = 10 nm, (right-top) $x_f \sim 0.28$, n-type, R_{rms} = 6.9 nm, (left-bottom) $x_f \sim 0.41$, n-type, R_{rms} = 9.5 nm, and (right-bottom) $x_f \sim 0.50$, n-type, R_{rms} = 4.2 nm. (b) FE-SEM images of ($Sn_{1-x_f}Pb_{x_f}$)S films with $x_f \sim 0.5$.

Surface morphology of $(Sn_{1-x_f}Pb_{x_f})S$ films was investigated by AFM and FE-SEM as shown in **Figure S2**. The film with x_f of ~ 0.08 showed p-type conduction, all the other three films showed n-type conduction. Although some surface roughness up to ~10 nm is found, uniform film microstructure and texture are confirmed. The FE-SEM images of $(Sn_{1-x_f}Pb_{x_f})S$ films with x_f ~ 0.5 also show the uniform contrast over the whole observation area and in the magnified image on right, showing that no Pb-rich segregation is detected.

3. Electron-probe microanalysis (EPMA) chemical composition mapping

Table S1 | Chemical composition mapping data for $(Sn_{1-x_f}Pb_{x_f})S$ film with x_f ~ 0.5.

**Data position schematic diagram**	**Data position**	**Sn (at. %)**	**Pb (at. %)**	**S (at. %)**	**Pb/(Sn+Pb)**	**S/(Sn+Pb)**
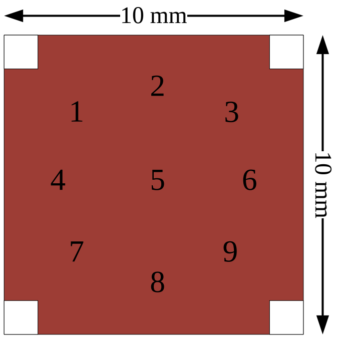	1	26.7	24.3	49.3	0.476	0.967
	2	26.7	24.2	49.1	0.475	0.965
	3	26.9	24.4	48.7	0.476	0.949
	4	26.8	24.6	48.6	0.479	0.946
	5	26.6	24.8	48.6	0.483	0.946
	6	26.7	25.1	48.2	0.485	0.931
	7	26.6	24.5	48.9	0.479	0.957
	8	26.8	24.7	48.5	0.480	0.942
	9	26.5	25.3	48.2	0.488	0.930

The chemical composition mapping of a $(Sn_{1-x_f}Pb_{x_f})S$ film with x_f ~ 0.5 was measured by EPMA as summarized in **Table S1**. We measured chemical compositions

at the uniformly-distributed 9 positions over the 10 × 10 mm^2 sample area. All the Pb/(Sn+Pb) and S/(Sn+Pb) ratios drop in the narrow regions 0.475−0.488 and 0.930−0.967, respectively, guaranteeing the uniform chemical composition.

4. Band alignment of *n*-type $(Sn_{0.5}Pb_{0.5})S$ / *p*-type Si pn junction.

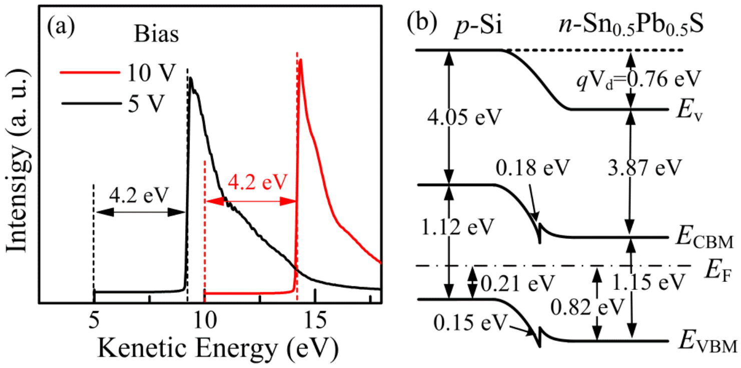

Figure S3 | Band alignment. (a) UPS spectrum from $(Sn_{0.5}Pb_{0.5})S$ film with bias voltages of 5 and 10 V. (b) Band alignment of *n*-type $(Sn_{0.5}Pb_{0.5})S$ / *p*-type Si pn junction.

Band alignment of *n*-$(Sn_{0.5}Pb_{0.5})S$ / *p*-Si pn heterjunction is shown in **Figure S3**b, where the work function of the $(Sn_{0.5}Pb_{0.5})S$ film (4.2 eV) was determined from the relative cutoff energy of the secondary electrons in the UPS He I spectrum shown in **Figure S3**a and the electron affinity of Si (4.05 eV) obtained from a literature [S1]. It gives the built-in potential (V_{bi}) of 0.76 eV, the conduction band offset of +0.18 eV, and the valence band offset of +0.15 eV (the positive sign indicates that the band edges are higher in the $(Sn_{0.5}Pb_{0.5})S$ layer). The V_{bi} roughly explains the experimental threshold voltage ~0.67 V (obtained by extrapolating the straight line region in **Figure 2**e).

5. Pb valence state examined by XPS.

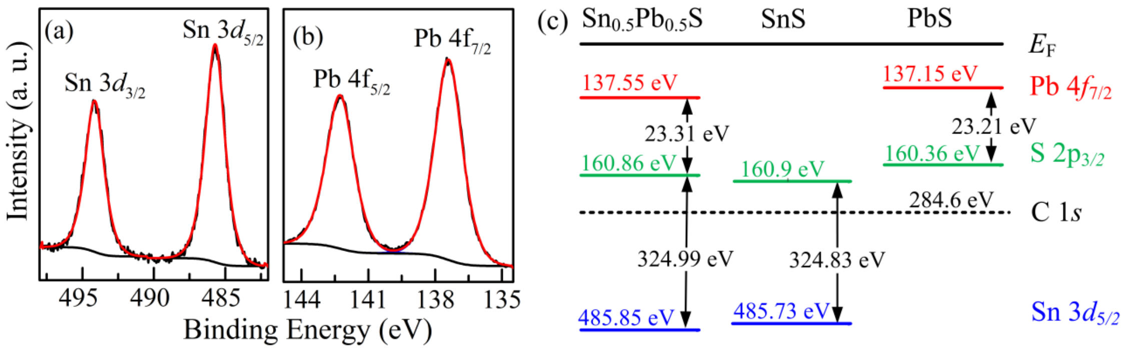

Figure S4 | XPS spectra. (a) Sn 3*d* and (b) Pb 4*f* core level XPS spectra of $(Sn_{0.5}Pb_{0.5})S$ film. (c) Energy alignment diagram for Sn 3*d*, Pb 4*f* and S 2*p* core levels of $(Sn_{0.5}Pb_{0.5})S$ film, pure SnS film, and PbS bulk sample.

XPS spectra were measured for $(Sn_{0.5}Pb_{0.5})S$ film, pure SnS film, and PbS bulk sample, where the samples were protected in an Ar atmosphere during the transfer from PLD preparation chamber/glove box to the XPS chamber. The binding energies were calibrated with reference to the C 1*s* level of adsorbed carbon-related molecules. Except for a little amount of OH, no other oxygen signal can be observed for all the samples. After cleaned by Ar^+ sputtering, the signal of OH disappeared, suggesting that the OH absorbed only on the surface. Sn 3*d* and Pb 4*f* core level spectra of the $(Sn_{0.5}Pb_{0.5})S$ film are shown in **Figures S4**a and b, respectively. The spectra were fitted using Gauss-Lorentz functions, and only one signal peak was obtained for each Sn 3*d* and the Pb 4*f* spectrum, indicating that the Sn and the Pb had single charge states, respectively.

The obtained energies of Sn 3*d*, Pb 4*f* and S 2p core levels of the $(Sn_{0.5}Pb_{0.5})S$ film, pure SnS film, and PbS bulk sample are aligned in the **Figure S**4c. The pure SnS film exhibited the Sn $3d_{5/2}$ peak at 485.73 eV, the energy difference between the Sn $3d_{5/2}$

peak and the S $2p_{3/2}$ peak (Δ_{Sn-S}) was about 324.83 eV. The Pb $4f_{7/2}$ peak of the PbS sample was centered at 137.15 eV, the difference between the Pb $4f_{7/2}$ peak and the S $2p_{3/2}$ peak (Δ_{Pb-S}) was 23.21 eV. These results are consistent with other reports on SnS and PbS samples [S2-S4]. For the $(Sn_{0.5}Pb_{0.5})S$ film, the Sn $3d_{5/2}$ peak was located at 485.85 eV with Δ_{Sn-S} of 324.99 eV, agreeing with those of the pure SnS. The Pb $4f_{7/2}$ peak was located at 137.55 eV, and Δ_{Pb-S} was 23.31 eV, very close to that of the PbS sample, suggesting that the Pb in the $(Sn_{0.5}Pb_{0.5})S$ film was of the +2 charge state.

References

S1. Kaiser, W. J., Bell, L. D., Hecht, M. H. & Grunthaner, F. J. Scanning tunneling microscopy characterization of the geometric and electronic structure of hydrogenterminated silicon surfaces. *Journal of Vacuum Science & Technology A* **6**, 519–523 (1988).

S2. Ettema, A. R. H. F. & Haas, C. An X-ray photoemission spectroscopy study of interlayer charge transfer in some misfit layer compounds. *J. Phys.: Condens. Matter* **5**, 3817–3826 (1993).

S3. Shalvoy, R. B., Fisher, G. B. & Stiles, P. J. Bond ionicity and structural stability of some average-valence-five materials studied by x-ray photoemission. *Phys. Rev. B* **15**, 1680–1697 (1997).

S4. Takahashi, M., Ohshima, Y., Nagata, K. & Furuta, S. Electrodeposition of PbS films from acidic solution. *J. Electroanal. Chem.* **359**, 281–286 (1993).
